# β-lactam-induced OMV release promotes polymyxin tolerance in *Salmonella enterica* sv. Typhi

**DOI:** 10.3389/fmicb.2024.1389663

**Published:** 2024-03-25

**Authors:** Pedro Marchant, Erika Vivanco, Andrés Silva, Jan Nevermann, Ignacio Fuentes, Boris Barrera, Carolina Otero, Iván L. Calderón, Fernando Gil, Juan A. Fuentes

**Affiliations:** ^1^Laboratorio de Genética y Patogénesis Bacteriana, Departamento de Ciencias Biológicas, Facultad de Ciencias de la Vida, Universidad Andres Bello, Santiago, Chile; ^2^Escuela de Tecnología Médica, Facultad de Salud, Universidad Santo Tomás, Santiago, Chile; ^3^Escuela de Química y Farmacia, Facultad de Medicina, Universidad Andres Bello, Santiago, Chile; ^4^Laboratorio de RNAs Bacterianos, Departamento de Ciencias Biológicas, Facultad de Ciencias de la Vida, Universidad Andres Bello, Santiago, Chile; ^5^Microbiota-Host Interactions and Clostridia Research Group, Universidad Andres Bello, Santiago, Chile

**Keywords:** outer membrane vesicles (OMV), *Salmonella enterica* serovar Typhi, polymyxin, beta lactam antibiotic, cross-resistance

## Abstract

The rise of multidrug-resistant bacteria is a global concern, leading to a renewed reliance on older antibiotics like polymyxins as a last resort. Polymyxins, cationic cyclic peptides synthesized nonribosomally, feature a hydrophobic acyl tail and positively charged residues. Their antimicrobial mechanism involves initial interaction with Gram-negative bacterial outer-membrane components through polar and hydrophobic interactions. Outer membrane vesicles (OMVs), nano-sized proteoliposomes secreted from the outer membrane of Gram-negative bacteria, play a crucial role in tolerating harmful molecules, including cationic peptides such as polymyxins. Existing literature has documented environmental changes’ impact on modulating OMV properties in *Salmonella* Typhimurium. However, less information exists regarding OMV production and characteristics in *Salmonella* Typhi. A previous study in our laboratory showed that *S.* Typhi Δ*mrcB*, a mutant associated with penicillin-binding protein (PBP, a β-lactam antibiotic target), exhibited hypervesiculation. Consequently, this study investigated the potential impact of β-lactam antibiotics on promoting polymyxin tolerance via OMVs in *S.* Typhi. Our results demonstrated that sub-lethal doses of β-lactams increased bacterial survival against polymyxin B in *S.* Typhi. This phenomenon stems from β-lactam antibiotics inducing hypervesiculation of OMVs with higher affinity for polymyxin B, capturing and diminishing its biologically effective concentration. These findings suggest that β-lactam antibiotic use may inadvertently contribute to decreased polymyxin effectivity against *S.* Typhi or other Gram-negative bacteria, complicating the effective treatment of infections caused by these pathogens. This study emphasizes the importance of evaluating the influence of β-lactam antibiotics on the interaction between OMVs and other antimicrobial agents.

## Introduction

*Salmonella enterica* subspecies *enterica* serovar Typhi (*S.* Typhi) is a strictly human-restricted *Enterobacteriaceae* responsible for typhoid fever, a severe systemic disease that affects over 20 million individuals worldwide, with approximately 200,000 annual fatalities, and particularly prevalent in developing countries (Ajibola et al., 2018; Bhutta et al., 2018; Johnson et al., 2018; Marchello et al., 2020). The infection initiates through the ingestion of contaminated water or food (Hook et al., 1990). Subsequently, the bacteria locate themselves in the distal ileum, facilitating internalization via intestinal epithelial cells or the M cells of the Peyer’s patches. Following this, *S.* Typhi infiltrates the underlying lymphoid tissue, where it is engulfed by dendritic cells, macrophages, or neutrophils, leading to transportation to deep organs such as the spleen, liver, and bone marrow, thereby triggering a systemic infection (Galan, 1996; Miao et al., 2003; Johnson et al., 2018). Moreover, it is noteworthy that approximately 5% of recovered patients continue to shed *S.* Typhi for extended periods through feces, remaining as asymptomatic chronic carriers (Gunn et al., 2014).

Typhoid fever mortality is 1%–4%, but this rate increases significantly to 10%–20% in the absence of antibiotic treatment (WHO, 2008). The emergence of multi-resistant strains in this context poses a severe problem, leading to a higher rate of disease recurrence (Kaushik et al., 2014; Als et al., 2018; Schwartz and Morris, 2018). Consequently, the prevalence of multidrug-resistant *S.* Typhi strains has necessitated the use of quinolones as a treatment approach (Parry et al., 2013; Karkey et al., 2018). However, the appearance of quinolone-resistant isolates has subsequently shifted the focus to azithromycin usage. In cases of more complicated infections or when azithromycin is not recommended, β-lactam antibiotics are employed (Pandit et al., 2007; Karkey et al., 2018). Notably, β-lactam antibiotics are widely used worldwide to treat bacterial infections, including those caused by *Enterobacteriaceae*, which has likely contributed to the emergence of resistant strains (Ahamed Riyaaz et al., 2018; Karkey et al., 2018; Cobos-Puc et al., 2020; De Angelis et al., 2020). Thus, a comprehensive study of antibiotic resistance in *Enterobacteriaceae*, including *S.* Typhi, is imperative for the development of new approaches to effectively treat or prevent bacterial diseases in the future (Neupane et al., 2021).

Antimicrobial resistance is undeniably a severe concern affecting humans, animals, and the environment. The emergence and dissemination of multidrug-resistant bacteria present a globally recognized issue. The primary source of resistant bacteria is likely linked to the improper use of antibiotics or other antimicrobial agents, enabling the selection and subsequent spread of genetic traits within the microbial community, exacerbating the situation (Aslam et al., 2018). As a result, the rise of multidrug-resistance pathogens, including *Enterobacteriaceae* and other bacterial species, poses a significant worldwide threat (Velkov et al., 2013; Jeannot et al., 2017; Srinivas and Rivard, 2017; Sherry and Howden, 2018; Sun et al., 2018).

Given the challenges in developing novel and effective antibiotics against multidrug-resistant bacteria, the revival of old antibiotics, especially polymyxins (colistin and polymyxin B), has garnered increasing attention (Velkov et al., 2013; Garg et al., 2017). Nevertheless, the use of polymyxins in standard therapies has been limited due to potential nephrotoxicity and neurotoxicity. However, considering the severity of infections caused by multidrug-resistant bacteria and the scarcity of viable therapeutic alternatives, polymyxins have resurfaced as a last line of action in these cases (Velkov et al., 2013; Sun et al., 2018).

Polymyxins are cyclic peptides synthesized nonribosomally, characterized by a hydrophobic acyl tail and positively charged residues, which confer remarkable antibiotic activity (Daugelavicius et al., 2000; Sun et al., 2018). Their antimicrobial mechanism involves an initial interaction with the outer-membrane components of Gram-negative bacteria, facilitated by polar and hydrophobic interactions (Daugelavicius et al., 2000; Sun et al., 2018). Given their cationic nature, polymyxins electrostatically interact with negatively charged biomolecules, primarily targeting the negatively charged lipid A moieties of lipopolysaccharides (LPS) located on the outer leaflet of the bacterial membrane, although interactions with proteins have also been reported (Daugelavicius et al., 2000; van der Meijden and Robinson, 2015; Sun et al., 2018). The integration of polymyxins into the outer membrane leads to a disruption in the packing of contiguous lipid A molecules, thereby compromising the permeability barrier and triggering an osmotic imbalance, ultimately culminating in bacterial death (Daugelavicius et al., 2000; Falagas and Kasiakou, 2006; Velkov et al., 2013; Trimble et al., 2016).

Currently, there is a growing body of literature addressing polymyxin resistance, with numerous reports being published (Sun et al., 2018; Li et al., 2019). Notably, mutations in chromosomal genes have been identified as key contributors to alterations in the bacterial envelope, particularly affecting the composition of LPS, thus inducing polymyxin resistance in various species (Sun et al., 2018; Li et al., 2019). These mutations result in a reduction of anionic charges within the bacterial envelope, specifically in the LPS, thereby diminishing the electrostatic binding of polymyxin to the bacterial outer membranes (Li et al., 2019). As most of these mutations are recessive in nature, the dissemination of polymyxin resistance has been predominantly linked to the acquisition of transferable *mcr* genes. The *mcr* genes, denoted as *mcr-1* to *mcr-10*, encode for a phosphoethanolamine transferase, which modulates the structure of the lipid A, leading to a decrease in its negative charge and consequently interfering with the interaction between polymyxins and the bacterial envelope (Olaitan et al., 2014). Despite significant progress in unraveling the mechanism underlying polymyxin resistance, further research in this domain remains imperative. In this context, outer membrane vesicles (OMVs) have gained increasing attention due to their capacity to mitigate the impact of antimicrobial agents targeting membranes (Grenier et al., 1995; Kobayashi et al., 2000; Manning and Kuehn, 2011; Kulkarni et al., 2015; Roszkowiak et al., 2019; Marchant et al., 2021).

Outer membrane vesicles (OMVs) are nano-sized proteoliposomes secreted from the outer membrane of Gram-negative bacteria (Kulp and Kuehn, 2010). OMV biogenesis primarily depends on (1) the accumulation of misfolded proteins in the periplasm, (2) changes in the LPS composition, or (3) the dissociation of the outer membrane in specific zones lacking proper attachments to underlying structures (e.g., when the peptidoglycan is weakened; Kulp and Kuehn, 2010; Kulkarni and Jagannadham, 2014; Nevermann et al., 2019). OMVs participate in various biological processes, enhancing bacterial fitness through offensive and defensive functions. They serve as vehicles for transporting and delivering virulence factors or act as bacterium-like baits that protect bacteria from bacteriophages and antimicrobial agents, among other essential functions (MacDonald and Kuehn, 2012; Schwechheimer and Kuehn, 2015; Gao and van der Veen, 2020; Rueter and Bielaszewska, 2020; Marchant et al., 2021). Regarding their defensive functions, OMVs play a significant role in tolerating diverse harmful molecules in different bacterial species. For instance, they contribute to the tolerance of toluene in *Pseudomonas putida*, chlorhexidine in *Porphyromonas gingivalis*, and polymyxins in *Escherichia coli* and *Acinetobacter baumannii* (Grenier et al., 1995; Kobayashi et al., 2000; Manning and Kuehn, 2011; Roszkowiak et al., 2019).

Typically, assessing OMVs as protective agents against polymyxin or other antimicrobial compounds involves extracting OMVs and introducing them to axenic reporter cultures to ascertain the extent of protection (Manning and Kuehn, 2011; Kulkarni et al., 2015; Roszkowiak et al., 2019). However, our recent findings shed light on a distinct perspective—we reported a functional transfer of OMV-mediated polymyxin tolerance from *S.* Typhi hypervesiculating mutants to susceptible bacteria within co-cultures, showing that the purification of OMVs was not required to observe the protection phenotype (Marchant et al., 2021). Moreover, the protection against polymyxin was not solely attributed to the hypervesiculating phenotype of *S.* Typhi mutants (Δ*degS* and Δ*tolR*, both involved in OMV biogenesis) but also to changes in OMV properties, which influenced the OMV Zeta potential, thereby enhancing the OMVs’ ability to sequester polymyxin (Marchant et al., 2021). It is crucial to acknowledge that not all mutants linked to hyper-vesiculation yield OMVs possessing protective attributes against polymyxin B, as observed in the case of the *S.* Typhi Δ*rfaE* mutant (Nevermann et al., 2019; Marchant et al., 2021). Therefore, hypervesiculation alone cannot serve as a reliable indicator of increased protection against polymyxin B.

Existing literature has documented the impact of environmental changes on modulating OMV properties in *Salmonella* Typhimurium (Orench-Rivera and Kuehn, 2016; Bonnington and Kuehn, 2017). However, considerably less information is available regarding the production of OMVs and their characteristics in *S.* Typhi. A previous study conducted in our laboratory showed that *S.* Typhi Δ*mrcB*, a mutant associated with penicillin-binding protein (PBP, a β-lactam antibiotic target), exhibited hypervesiculation (Nevermann et al., 2019). In this sense, we sought to explore the potential repercussions of the presence of β-lactam antibiotics in promoting tolerance to polymyxin in *S.* Typhi via OMVs.

This study shows that exposure to sub-lethal concentrations of β-lactam antibiotics enhances bacterial viability when challenged with polymyxin B in *S.* Typhi. This phenomenon is elucidated by the propensity of β-lactam antibiotics to induce hypervesiculation of OMVs, which exhibit an augmented affinity for polymyxin B compared to OMVs generated in the absence of β-lactam antibiotics. As a result, the amount of polymyxin B available to eradicate bacteria is reduced. This study underscores the importance of assessing the impact of β-lactam antibiotics on the interplay between OMVs and other antimicrobial agents.

## Materials and methods

### Bacterial strains, media, and culture conditions

*Salmonella enterica* subsp. *enterica* sv. Typhi strain STH2370 (*S.* Typhi) was used as the parental strain (Valenzuela et al., 2014). The strain was routinely grown in liquid culture using Luria Bertani medium (Bacto tryptone [Gibco], 10 g/L; Bacto yeast extract [Gibco], 5 g/L; NaCl [Winkler], 5 g/L; prepared in distilled water) at 37°C with shaking. When required, the medium was supplemented with agar (15 g/L), ampicillin (Amp, AppliChem GmbH), polymyxin B sulfate (Pmb, AppliChem GmbH), or meropenem (Mer, Sigma Aldrich). For *S.* Typhi, 1× Amp represents a concentration equivalent to the Minimum Inhibitory Concentration (MIC) of Amp (6.25 μg/mL), while 1× Pmb corresponds to the MIC of polymyxin B (0.31 μg/mL). Various concentrations based on these standards were employed, such as 0.25× Amp (1.56 μg/mL), 0.5× Amp (3.13 μg/mL), 2× Pmb (0.63 μg/mL), and 4× Pmb (1.25 μg/mL).

### OMV isolation, quantification, and size measurement

OMVs were isolated as previously described (Liu et al., 2016; Nevermann et al., 2019; Marchant et al., 2021). To that end, bacteria were cultured in LB (or LB supplemented with antibiotics) at 37°C with shaking until reaching an optical density at 600 nm (OD_600_) of 1.1–1.3. OMVs were stored at −20°C until use. OMV yield was quantified by determining the protein content using a BCA assay and/or the lipid content with the FM4-64 molecular probe and standardizing with the CFU, as reported (McBroom et al., 2006; Deatherage et al., 2009).

The following method was utilized to calculate the relative quantity of OMVs. Equal amounts of LB medium and LB medium with sub-lethal concentrations of ampicillin were cultured as described above. The CFU/mL per plating were calculated, and their respective OMVs were isolated and suspended in 1 mL of DPBS. Equal volumes underwent TEM to count OMVs in each field. The count of OMVs was divided by the field width (μm) to establish OMVs μm^−1^, which were further divided by the corresponding CFU mL^−1^, resulting in OMVs mL μm^−1^ CFU^−1^. At least 6 different fields for each biological replicate were counted. Relative quantification was conducted compared to the OMVs obtained from LB without ampicillin.

We determined OMV size as described (Deatherage et al., 2009; Nevermann et al., 2019), with results presented as the diameter.

### Determination of minimal inhibitory concentration

Minimal inhibitory concentration (MIC) was determined using a broth dilution method with modifications as described (Cuenca-Estrella et al., 2003). Briefly, bacteria were cultured as detailed above. Microorganisms were diluted in phosphate-buffered saline (PBS) (Gibco) (0.5 McFarland) and then further diluted (1000-fold) in LB before seeding a 96-well plate. Each well received 180 μL of this dilution, along with a solution of 10 μL containing Pmb or Amp, and 10 μL of PBS. When indicated, the 10 μL of PBS was replaced by 10 μL of purified OMVs to achieve a final known concentration. Alternatively, and when indicated, the 10 μL PBS was replaced by 10 μL of bacterial supernatant. To obtain the bacterial supernatant, bacteria were cultured in LB (or LB supplemented with antibiotics) as stated above (OD_600_ = 1.1–1.3), centrifuged for 10 min at 5,400 × *g* at 4°C, the pellet was discarded, and the supernatant fraction was filtered (0.45 μm). The 96-well plates were incubated overnight at 37°C, and MIC was determined by OD_600_ measurement, corroborated by visual inspection, and validated by plating onto agar plates.

### Determination of doubling time

Bacteria were cultured in LB (or LB supplemented with antibiotics) as described above, and OD_600_ was recorded every 10 min to construct a growth curve. To calculate μ and t_d_, we used the following:


$$\mu =\frac{\ln\left(N\right)-\ln\left(N_{0}\right)}{t-t_{0}}\;t_{d}=\frac{0.693}{\mu }\times 60$$

Where *μ* (h^−1^): growth rate; *N*: bacteria at the end of the logarithmic phase (OD_600_); *N_0_* bacteria at the beginning of the logarithmic phase (OD_600_); *t*: time at the end of the logarithmic phase (h); *t_0_*: time at the beginning of the logarithmic phase (h); *t_d_*: doubling time (min).

### Transmission electron microscopy

OMV extracts were bound to formvar-coated slot grids, stained with 1% aqueous uranyl acetate for 1 min, and viewed with a Philips Tecnai 12 (Biotwin) transmission electron microscope, as described (Nevermann et al., 2019; Marchant et al., 2021).

### SDS-page

As previously reported (Nevermann et al., 2019), OMVs (corresponding to 50 μg of proteins) were resolved by SDS-PAGE and visualized with a One-Step Blue (Biotium).

### Determination of Zeta potential

The Zeta potential of OMVs was measured at room temperature (25°C) using a Zetasizer Nano series MPT-Z multi-Purpose Titrator (Malvern, United Kingdom). The device was equipped with a Helium-Neon laser (633 nm), serving as a light source. Zetasizer measurements in aqueous media were conducted at a detection angle of 173.13°, with a measurement range covering diameters from 0.3 nm to 10 μm. Capillary cells DTS 1071 were employed for the measurements. Pmb and OMVs were resuspended in ultrapure water for Zeta potential measurements. In this study, each measurement was conducted with a minimum of 50 events and up to a maximum of 100 events, contingent upon the quality of the results as determined by the Malvern Zetasizer software (version 7.11) calculations. Subsequently, 10 measurements were taken for each sample, resulting in a total range of 500 to 1,000 measurements per sample. Additionally, at least three independent biological replicates were performed for every measurement. The measurements provide insights into the surface charge characteristics of OMVs and the impact of Pmb on their Zeta potential, as previously reported (Marchant et al., 2021).

### Estimation of polymyxin sequestration by OMVs

In order to assess Zeta potential changes resulting from the interaction with Pmb, OMV extracts were prepared at a concentration of 50 μg/mL. The OMV extracts were then mixed with varying concentrations of Pmb (0, 5, 50, or 100 μg/mL) and incubated for 30 min at 37°C with gentle agitation. Subsequently, the mixture underwent ultrafiltration using an Ultracel® 100 kDa ultrafiltration column (Amicon® Bioseparations) at 5,400 × *g* for 10 min to remove unbound Pmb. The ultrafiltrate obtained with 100 μg/mL Pmb was reserved for further analysis. To evaluate the impact on Zeta potential, OMVs were resuspended in one volume of ultrapure water before measurement. Control measurements included the Zeta potential of water alone (−0.05 ± 0.35 mV), water +100 μg/mL Pmb (4.45 ± 0.33 mV), and water +100 μg/mL Pmb that was ultrafiltered and subsequently resuspended in one volume of ultrapure water (0.51 ± 0.22 mV). These controls provided a baseline for assessing the effects of Pmb on OMVs. To estimate the relative amount of Pmb sequestered by OMVs, the reserved ultrafiltrate was diluted 10 times in LB and then serially diluted in LB to determine the last dilution that inhibited the growth of *S.* Typhi. A control solution with no OMVs was used for comparison in the growth inhibition assay.

## Results

### The presence of a β-lactam antibiotic (Amp) increases bacterial survival against polymyxin B

**Figure 1:**
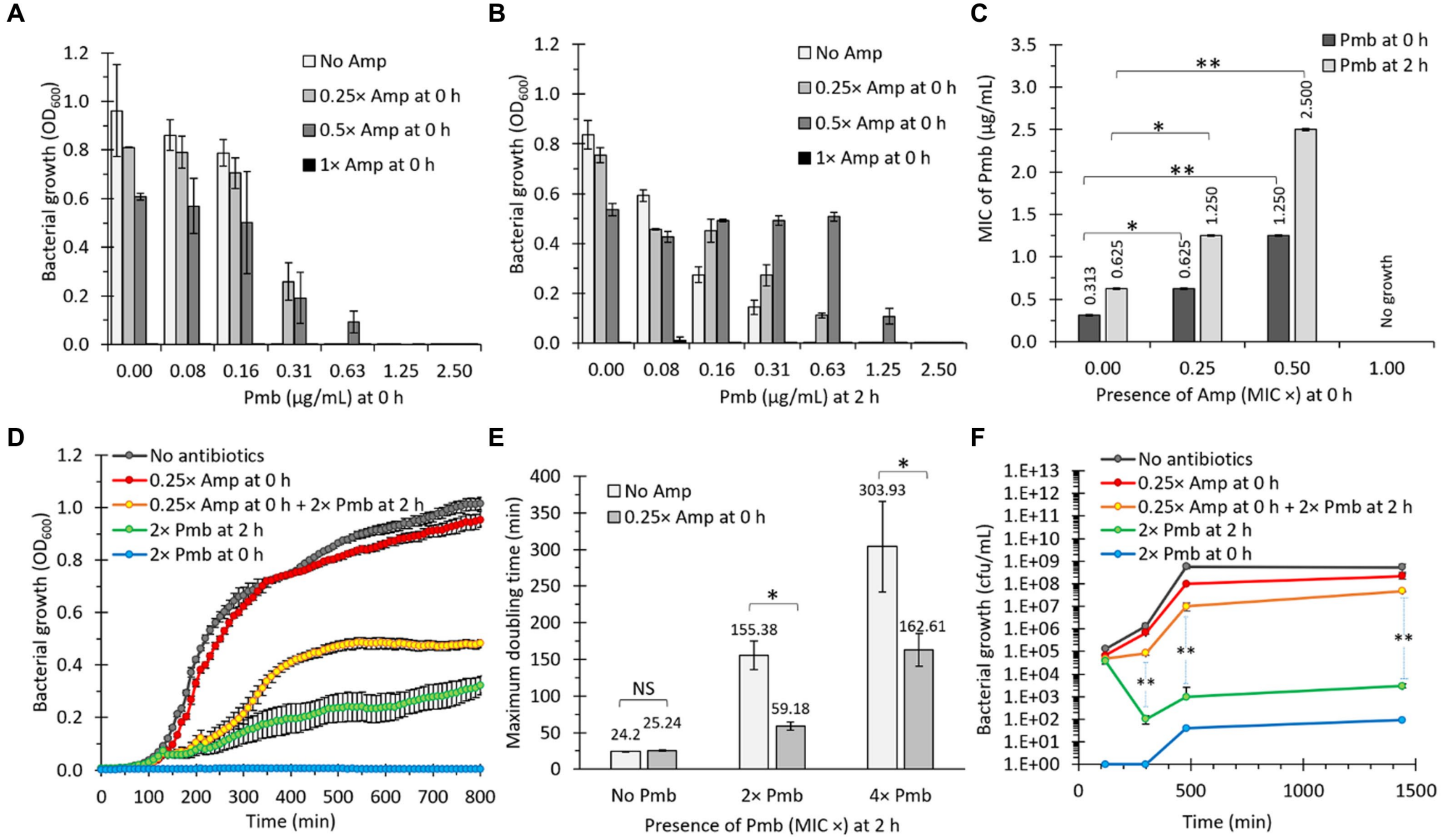
The presence of a β-lactam antibiotic (ampicillin, Amp) increases bacterial survival against polymyxin B (Pmb). **(A)** *Salmonella* Typhi was incubated with different concentrations of Amp and Pmb prior to determining bacterial growth as absorbance (OD_600_). Amp and Pmb were added simultaneously to culture media (0 h). *n* = 3, bars represent the standard error. **(B)** Same as in panel **(A)**, but Amp was added first (0 h), then bacteria were incubated for 2 h at 37°C before adding Pmb. **(C)** MIC of Pmb for *S.* Typhi in the presence of Amp. Dark bars: Amp was added simultaneously with Pmb (0 h). Light bars: After adding Amp (0 h), bacteria were cultured for 2 h at 37°C before adding Pmb. *n* = 3, bars represent the standard error. The statistical analysis involved one-way ANOVA followed by Tukey’s *post hoc* test. **(D)** Growth curves (OD_600_) of *S.* Typhi in LB. When indicated, Amp was supplemented at 0 h, and Pmb was supplemented at either 0 h or 2 h. n = 30, bars represent the standard error. **(E)** Maximum doubling time of *S.* Typhi in LB. When indicated, Amp was supplemented at 0 h, and Pmb was supplemented at 2 h. *n* = 30, bars represent the standard error. In this case, the exponential phase was analyzed. Welch’s t-test for unequal variances was used between the same Pmb concentration treatments. ^*^p < 0.05. **(F)** Growth curves (CFU/mL) of *S.* Typhi in LB. When indicated, Amp was supplemented at 0 h, and Pmb was supplemented at either 0 or 2 h. *n* = 9, bars represent the standard error. Welch’s *t*-test for unequal variances was used between 2× Pmb at 2 h and 0.25× Amp at 0 h + 2× Pmb at 2 h. ^*^p < 0.05 and ^**^p < 0.01 for all the figures. In all cases, 1× Amp is a concentration equivalent to the MIC of ampicillin, i.e., 6.25 μg/mL, whereas 1× Pmb is a concentration equivalent to the MIC of polymyxin B, i.e., 0.31 μg/mL. Other concentrations based on these standards were also used (e.g., 0.25× Amp: 1.56 μg/mL, 0.5× Amp: 3.13 μg/mL, 2× Pmb: 0.63 μg/mL, or 4× Pmb: 1.25 μg/mL). We observed similar results using meropenem (Mer) instead of Amp (data not shown).

During our laboratory investigations, we made a counter-intuitive observation: treatment of bacteria with sub-lethal concentrations of ampicillin (Amp) resulted in a heightened survival response when exposed to polymyxin B (Pmb), as opposed to untreated bacteria. To comprehensively explore this phenomenon, we systematically evaluated bacterial growth of *S.* Typhi upon exposure to increasing concentrations of Amp [with 1× Amp corresponding to the minimum inhibitory concentration (MIC): 6.25 μg/mL] in the presence of Pmb. As anticipated, bacterial growth was inhibited in the presence of 1× Amp. However, when exposed to sub-lethal concentrations of Amp (i.e., below 1× Amp), the bacteria exhibited the ability to grow in higher concentrations of Pmb compared to bacteria growing without Amp (Figure 1A, see with 0.31 and 0.63 μg/mL Pmb). Notably, this effect was more pronounced when the bacteria were pre-incubated with Amp for 2 h at 37°C before adding Pmb (Figure 1B).

The additional incubation period was expected to promote bacterial division, thereby increasing their survival to Pmb. It is noteworthy that Pmb has a propensity to adhere to biological membranes, resulting in a decrease in its effective concentration with increasing bacterial concentration (Velkov et al., 2013; Roszkowiak et al., 2019; Marchant et al., 2021). This phenomenon could explain the elevated MIC observed after 2 h of incubation in the absence of Amp (from 0.31 to 0.63 μg/mL, as depicted in Figure 1C). However, observed bacterial growth, quantified by the OD_600_, tends to correlate positively with sub-lethal concentrations of Amp as the Pmb concentration increases (Figure 1B), suggesting Amp’s contributory role in bacterial proliferation in the presence of Pmb. Consistently, the MIC to Pmb also positively correlates with Amp’s sublethal concentrations (Figure 1C). To further characterize this phenomenon, we conducted growth curve analyses measuring OD_600_ over time. As depicted in Figure 1D, adding 0.25× Amp at 0 h had almost negligible effects on bacterial growth compared to LB (no antibiotics), as evidenced by the similarity between the gray and red curves. In contrast, treatment with 2× Pmb (added at 0 h) inhibited growth (blue curve). Conversely, bacterial growth was observed when 2× Pmb was added after 2 h of incubation (green curve in Figure 1D), aligning with observations in Figure 1C. On the other hand, in bacteria pre-exposed to 0.25× Amp, the presence of 2× Pmb at 2 h consistently exerted a less deleterious effect compared with the treatment with 2× Pmb alone at 2 h (compare green and yellow curves, Figure 1D). This paradoxical finding suggests that the combined presence of two antibiotics, Amp and Pmb, fosters better bacterial growth than Pmb alone.

To quantify the protective effect conferred by Amp, we compared the maximum doubling time of cultures with and without 0.25× Amp, supplemented with Pmb (2× and 4×) at 2 h. The results revealed that a sub-lethal concentration of Amp decreased the doubling time by 2.6-fold in the presence of 2× Pmb and by 1.9-fold with 4× Pmb (Figure 1E). To validate these findings independently, we replicated the experiment illustrated in Figure 1D, assessing bacterial growth by quantifying colony-forming units (CFU), yielding highly congruent results (Figure 1F). Cumulatively, this evidence substantiates that the presence of Amp enhances bacterial growth in media containing Pmb.

### OMVs from bacteria treated with a sublethal concentration of Amp increase the survival of *Salmonella* Typhi against Pmb

**Figure 2:**
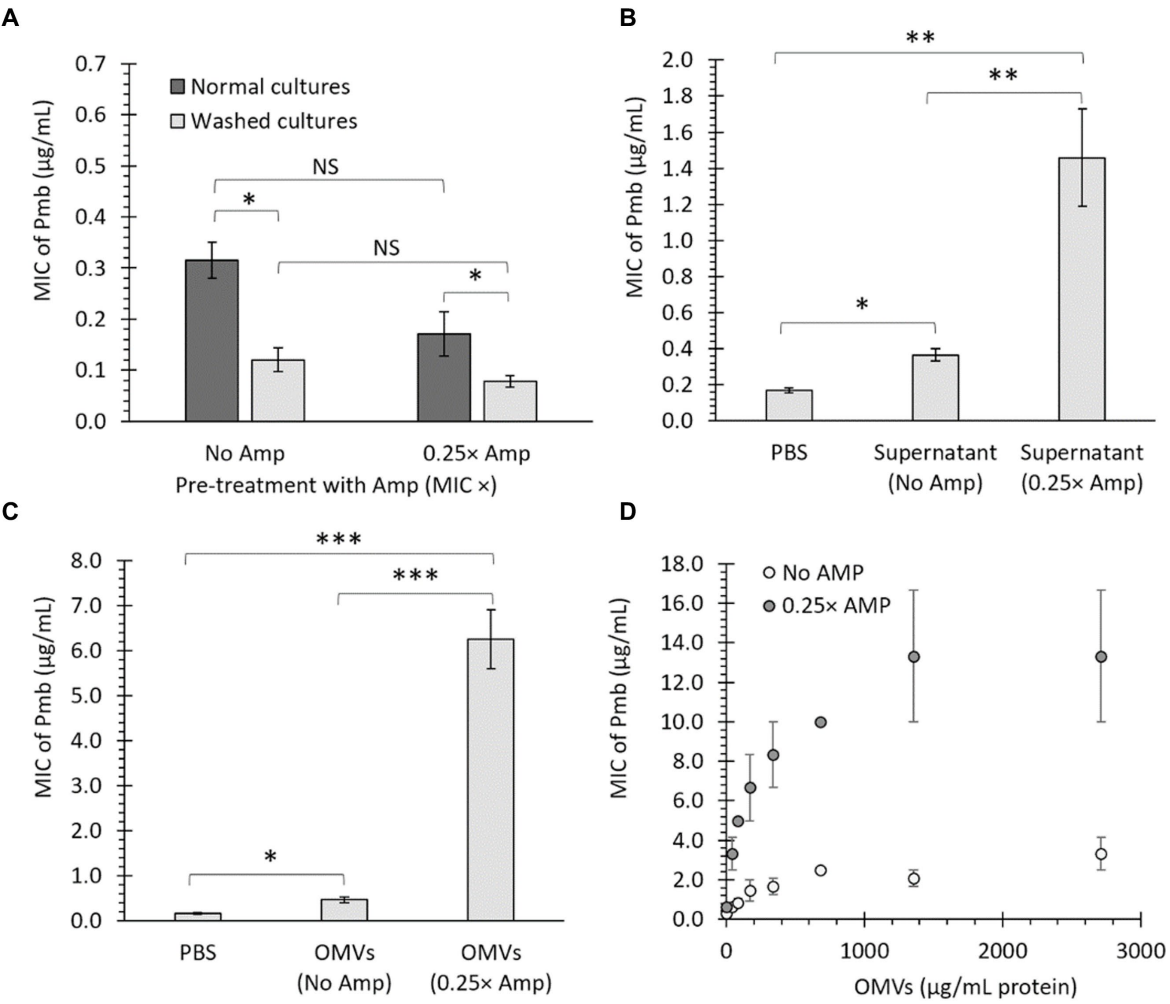
OMVs from bacteria treated with a sub-lethal ampicillin concentration (Amp) increase the survival of *Salmonella* Typhi against polymyxin B (Pmb). **(A)** MIC of Pmb after culturing *S.* Typhi in LB with either No Amp or 0.25× Amp. The bacteria were grown to the stationary phase (OD_600_ = 1.1 at 37°C with shaking) and tested immediately (dark bars) or washed thrice with LB (light bars) before testing the MIC of Pmb. The standard error is indicated for each treatment. The experiment was conducted with *n* = 6, and a two-way ANOVA with a Tukey’s *post hoc* test was performed. **(B)** MIC of Pmb for *S.* Typhi supplemented with bacterial supernatant. The supernatant was obtained from *S.* Typhi cultured in LB (No Amp) or LB supplemented with ampicillin (0.25× Amp) to stationary phase (OD_600_ = 1.1 at 37°C with shaking). As a control, PBS was used instead of supernatant. The supernatant (or PBS) was added to a washed culture of *S.* Typhi at a 5% v/v LB before determining the MIC. The experiment was conducted with n = 6, and statistical analysis was performed using a one-way ANOVA with Tukey’s *post hoc* test. **(C)** MIC of Pmb for *S.* Typhi supplemented with OMVs. The OMVs were extracted from *S.* Typhi cultured in LB (No Amp) or LB supplemented with Amp (0.25× Amp). OMVs, obtained from a 40 mL culture medium and resuspended in 1 mL of PBS, were added to *S.* Typhi at a 5% v/v in LB before determining the MIC. As a negative control, PBS was used instead of OMVs. The experiment was conducted with *n* = 6, and statistical analysis was performed using a one-way ANOVA with Tukey’s *post hoc* test. **(D)** MIC of Pmb for *S.* Typhi was evaluated in the presence of different known, final concentrations of OMVs (determined as the protein content). The experiment was conducted with *n* = 3; bars represent the standard error. For all cases, 0.25× Amp is a concentration equivalent to one-fourth of the MIC of ampicillin, i.e., 1.56 μg/mL. ^*^p < 0.05, ^**^p < 0.01, ^***^p < 0.001. NS, not statistically significant. We observed similar results using meropenem (Mer) instead of Amp (data not shown).

To elucidate the causative factors underlying Amp-dependent heightened growth in Pmb, with specific emphasis on genetic alterations (e.g., mutations), we subjected *S.* Typhi to culture conditions involving the presence or absence of Amp, in the absence of Pmb. Cultures were allowed to reach the stationary phase before ascertaining the MIC of Pmb. This approach aimed to investigate the possible accumulation of Pmb-tolerant mutants in the presence of Amp. Contrary to our expectations, the culture treated with Amp exhibited a slight, but not statistically significant, decrease in MIC to Pmb (Figure 2A, compare the dark bars). We attributed this result to the presence of ampicillin traces in the culture, which could impair bacterial growth and give confusing results. This inhibitory growth effect caused by antibiotics (including β-lactam antibiotics) is notorious when a lower concentration of bacteria is used as inoculum (Kaplan and Koch, 1964; Frenkel et al., 2021), a result of the necessary dilution inherent in the MIC determination assay. Thus, in order to eliminate any potential bias from the presence of ampicillin traces in the culture, bacterial samples were washed three times with LB before determining MIC. Figure 2A shows that the cleansed cultures (compare pale bars) displayed no differences when determining the MIC of Pmb, regardless of Amp’s presence or absence in the culture, ruling out that the accumulation of mutants could cause increased tolerance to Pmb. Nevertheless, washed cultures demonstrated a decrease in MIC to Pmb compared to non-washed cultures (compare dark and pale bars), suggesting a possible secreted factor contributing to enhanced survival against Pmb. The findings suggested that a secreted factor, rather than genetic alterations or mutations, was responsible for the heightened growth and enhanced survival against Pmb in the washed cultures.

We reasoned that if the factor responsible for protection against Pmb is a removable element that can be washed away, it should also be able to transfer its protective traits to previously washed cultures. In this context, we grew *S.* Typhi until reaching the stationary phase (OD_600_ = 1.1–1.3 at 37°C with shaking), washed the cultures thrice with LB, and added 1 volume of LB supplemented with 5% v/v supernatant obtained from stationary cultures grown without ampicillin (No Amp) or from cultures exposed to sub-lethal concentrations of ampicillin (0.25× Amp). As a control, we replaced the supernatant with PBS. As shown in Figure 2B, the inclusion of bacterial supernatant notably raises the MIC of Pmb, particularly when using supernatant from bacteria previously cultured with sub-lethal doses of ampicillin. This suggests that a secreted factor can transfer its protective traits to previously washed cultures and enhance their tolerance against Pmb.

We previously found that OMVs extracted from some *S.* Typhi mutants displayed enhanced capacity to trap Pmb (i.e., OMVs from Δ*tolR* and Δ*degS* mutants) compared to OMVs from the *S.* Typhi WT (Marchant et al., 2021). This effect was most pronounced when bacteria were incubated for 2 h before the exposure to Pmb, allowing the release and accumulation of OMVs (Marchant et al., 2021). In this sense, the observed boost in survival against Pmb with a sub-lethal Amp concentration, shown in Figure 1, could be linked to the secretion of OMVs. To investigate whether the defense against Pmb enhanced by Amp can be associated with OMVs, *S.* Typhi was cultured in LB (40 mL, without Amp) or LB with ampicillin (40 mL, 0.25× Amp) until reaching OD_600_ = 1.1 at 37°C with shaking before collecting an OMV suspension (1 mL) from each condition. Thus, *S.* Typhi cultured to stationary phase was washed three times with LB and then suspended in 1 volume of LB supplemented by 5% v/v of an OMV suspension from LB (OMVs No Amp), an OMV suspension from LB with ampicillin (OMVs 0.25× Amp), or PBS instead of OMVs before determining the MIC of Pmb. The data presented in Figure 2C indicates that including purified OMVs increases the MIC of Pmb in washed bacteria (PBS). Notably, OMVs derived from cultures with a sub-lethal concentration of ampicillin (0.25 × Amp) resulted in a higher MIC of Pmb than OMVs obtained from cultures without ampicillin (No Amp). These findings suggest that sub-lethal concentrations of β-lactam antibiotics result in (1) enhanced protective capabilities of OMVs against Pmb, and/or (2) hypervesiculation, leading to an augmented production of OMVs, as previously observed in the *S.* Typhi Δ*tolR* mutant (Marchant et al., 2021).

As a next step, OMVs were assessed for their ability to offer protection against Pmb in a concentration-dependent manner, following the method described in previous studies (Marchant et al., 2021). Figure 2D illustrates that OMVs derived from *S.* Typhi cultured in LB with No Amp provided increasingly effective protection against Pmb as the concentration of OMVs rose. Notably, when OMVs obtained from *S.* Typhi cultured in LB with a sub-lethal Amp concentration were used, they demonstrated significantly improved dose-dependent protection against PmB compared to OMVs extracted from cultures without Amp (showing between 2 and 6.4 times more pronounced protective effects). These findings indicate that OMVs generated in the presence of sub-lethal amounts of β-lactam antibiotics demonstrate enhanced abilities to offer protection against Pmb, compared to OMVs acquired without the presence of β-lactam antibiotics.

### Characterization of OMVs produced in media supplemented with sublethal amounts of β-lactam antibiotics

**Figure 3:**
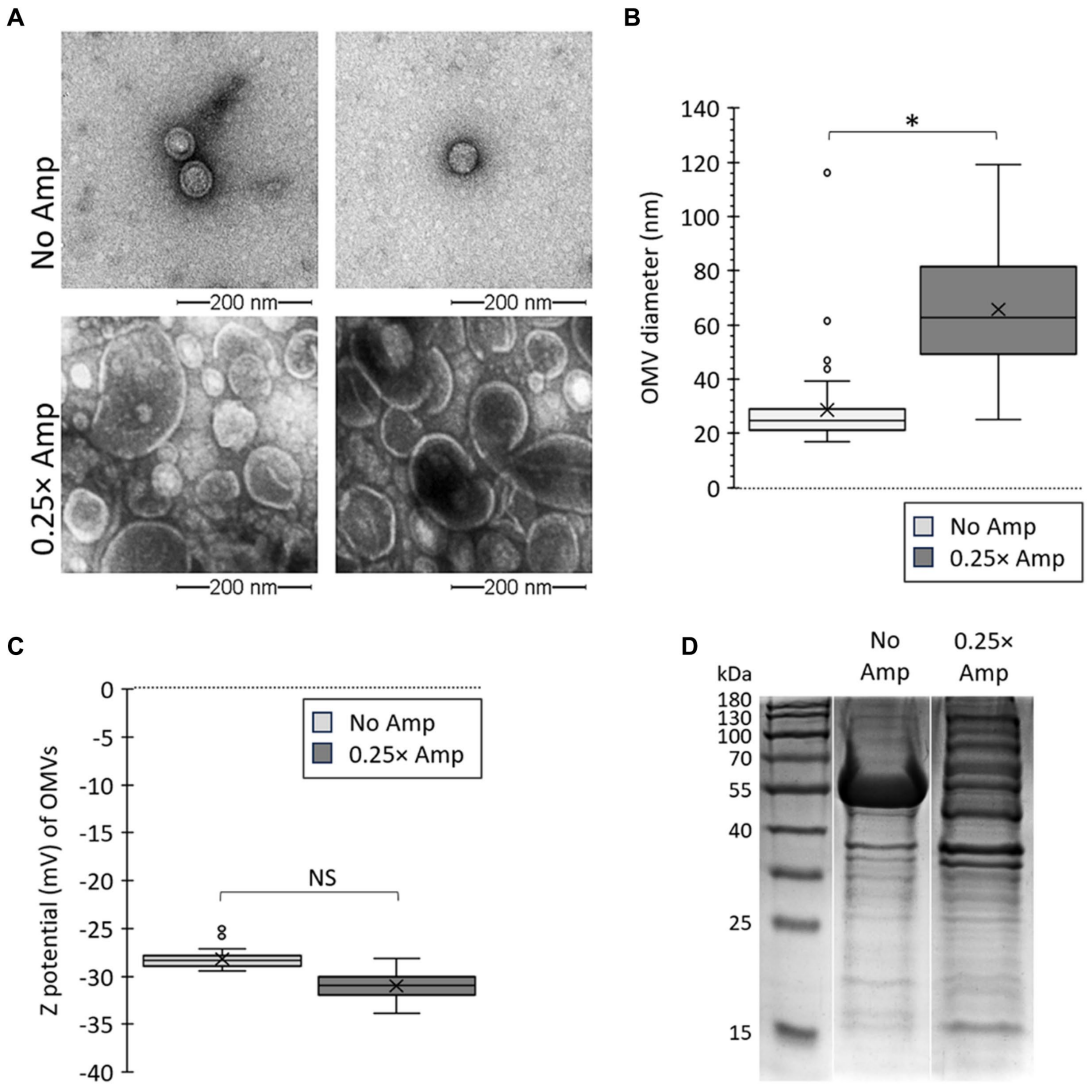
Sub-lethal concentrations of β-lactam antibiotics affect OMV size and Zeta potential. **(A)** *Salmonella* Typhi was cultured on LB (No Amp) or LB supplemented with sub-lethal concentrations of ampicillin (0.25× Amp) to OD_600_ = 1.1 before extracting OMVs. OMV extracts were observed by transmission electron microscopy (TEM). The bar corresponds to 200 nm. A representative experiment is shown (*n* = 3). **(B)** OMVs size determined as described (Nevermann et al., 2019; Marchant et al., 2021). OMVs were obtained from cultures similar to those described in **(A)**. OMVs analysis involved conducting Welch’s t-test for unequal variances, with 3 biological replicates, totaling the analysis of more than 300 OMVs for each condition. **(C)** Z potential (mV) of OMVs. OMVs were obtained from cultures similar to those described in panel **(A)**. OMVs analysis involved conducting Welch’s *t*-test for unequal variances, with 3 biological replicates and 50 to 100 technical replicates each time, totaling the analysis of 30,000 OMVs for each condition. For panels **(B,C)**, the symbol × represents the media, while dots represent atypical values. ^*^p < 0.05. For all cases, 0.25× Amp is a concentration equivalent to one-fourth of the MIC of ampicillin, i.e., 1.56 μg/mL. **(D)** Protein profile of OMVs obtained by culturing bacteria in LB (No Amp) or LB supplemented by sub-lethal concentrations of ampicillin (0.25× Amp). OMV proteins (50 μg/mL) were resolved by SDS-PAGE of OMV extracts. Proteins were visualized by One-Step Blue staining.

In order to further explore the impact of sublethal levels of β-lactam antibiotics on OMV production, we conducted a thorough analysis of the OMVs generated in media containing Amp. The OMVs from bacteria cultured in LB with sub-lethal doses of Amp were characterized and compared against those from bacteria grown in LB without any antibiotics using transmission electron microscopy (TEM). Our findings, depicted in Figure 3, indicate that sub-lethal concentrations of β-lactam antibiotics influenced the biogenesis of OMVs, as evidenced by changes in size (Figure 3A) and size distribution (Figure 3B). The Zeta potential of OMVs has also been evaluated. This parameter indicates the electrostatic potential at a particle’s slipping plane and is affected by the surface composition and charge of the particle. In the context of OMVs, assessing Zeta potential is crucial for identifying variations in cargo due to differences in charged molecules. Moreover, considering the Zeta potential is significant when examining interactions between bacterial cells and other components, particularly cationic elements like polymyxins (Drulis-Kawa et al., 2009; Halder et al., 2015; Roszkowiak et al., 2019; Marchant et al., 2021). Therefore, in our study, we analyzed the Zeta potential of OMVs produced in the presence of sublethal amounts of β-lactam antibiotics. Figure 3C depicts no notable difference in the Zeta potential of OMVs obtained with no ampicillin (No Amp) and sub-lethal concentrations of Amp (0.25× Amp).

Treating bacteria with sub-lethal concentrations of Amp was found to impact OMV biogenesis in *S.* Typhi, increasing the size and yield of the OMVs. Given that a significant portion of OMV cargo selection occurs during OMV biogenesis, and because of the impact of β-lactam antibiotics on this process, we examined the potential influence of β-lactam antibiotics on OMV protein cargo. Polymyxins demonstrate their antimicrobial effects by interacting with outer-membrane components, including proteins (Daugelavicius et al., 2000; van der Meijden and Robinson, 2015; Sun et al., 2018). Previously, it has been proposed that alterations in these components may account for the enhanced protective capabilities of OMVs against polymyxins in specific *S.* Typhi mutants (Marchant et al., 2021). Thus, we contrasted the protein composition of OMVs collected without antibiotics (No Amp) with those obtained with sub-lethal levels of β-lactam antibiotics (0.25× Amp). As shown in Figure 3D, the OMVs gathered when exposed to ampicillin exhibited a distinct protein profile compared to those acquired with LB alone, indicating the influence of the β-lactam antibiotic on OMV biogenesis. Altogether, these findings indicate that sub-lethal quantities of β-lactam antibiotics impact the formation and properties of OMVs in *S.* Typhi.

**Figure 4:**
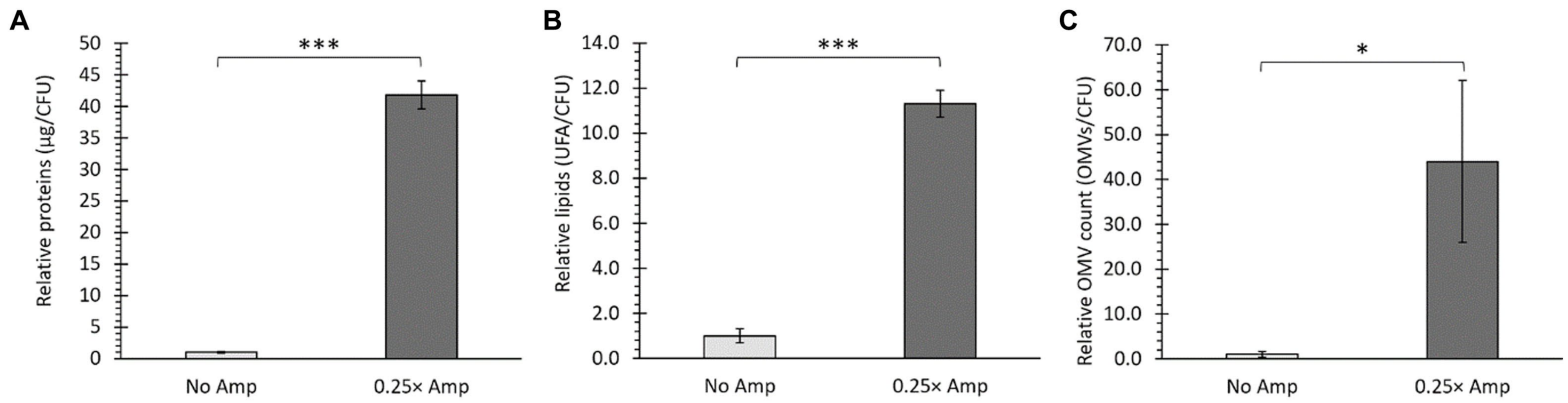
Sub-lethal doses of β-lactam antibiotics affected the production of OMVs. *Salmonella* Typhi was cultured in LB without ampicillin (No Amp) or with sub-lethal ampicillin concentrations (0.25× Amp) to OD_600_ = 1.1 before harvesting OMVs. Quantification of protein content **(A)**, lipid content **(B)**, and relative OMV count normalized to CFU **(C)** indicated increased OMV yield, as confirmed by *t*-test analysis (^*^p < 0.05, ^***^p < 0.001, *n* = 3). For all cases, 0.25× Amp is a concentration equivalent to one-fourth of the MIC of ampicillin, i.e., 1.56 μg/mL.

According to our findings, it appears that β-lactam antibiotics may enhance the production of OMVs. To investigate this hypothesis, we measured the OMV yield by evaluating the protein and lipid content of OMVs, which are commonly used indicators of abundance. These measurements were normalized with CFU in each case, as previously reported (McBroom et al., 2006; Deatherage et al., 2009; Marchant et al., 2021). Exposure to sublethal levels of β-lactam antibiotics led to notably elevated OMV production, as inferred by increased protein (Figure 4A) and lipid (Figure 4B) content compared to the control group. Furthermore, we estimated the relative OMV/CFU count, as described in Materials and Methods, obtaining the same conclusions (Figure 4C). The results align with those seen in the TEM (Figure 3A), where a greater quantity of OMVs released by the bacteria treated with β-lactam antibiotics was observed. Thus, we concluded that the presence of β-lactam antibiotics leads to increased OMV production in *S.* Typhi. Based on our findings, the existence of β-lactam antibiotics triggers heightened production of OMVs in *S.* Typhi, leading to a greater yield of these vesicles. This outcome suggests that the augmented protective capability of OMVs derived from culturing bacteria with sublethal doses of ampicillin stems not only from an increased quantity but also from generating OMVs with enhanced defensive properties, as inferred from Figure 2C.

### OMVs generated in the presence of sublethal concentrations of β-lactam antibiotics sequester Pmb

**Figure 5:**
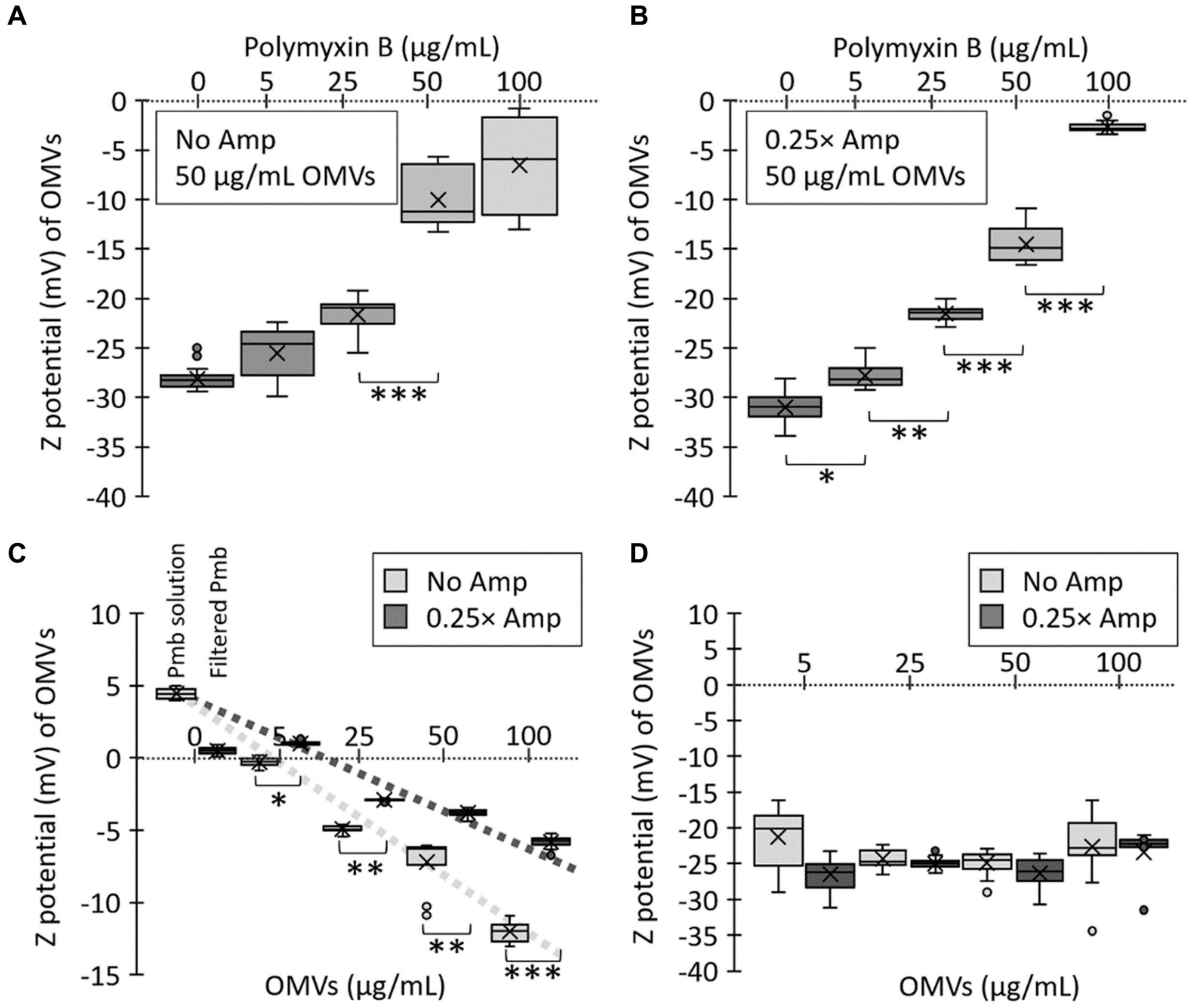
Polymyxin B (Pmb) interacts with OMVs from *Salmonella* Typhi, depolarizing their Zeta potential. Various concentrations of Pmb were combined with 50 μg/mL OMVs obtained from *S.* Typhi cultured in LB (No Amp) **(A)** or LB supplemented with a sub-lethal concentration of ampicillin (0.25× Amp). **(B)** The mixture underwent a 30-min incubation at 37°C, followed by ultrafiltration to eliminate unbound Pmb before Zeta potential determination. **(C)** Various concentrations of OMVs (assessed by the protein content) were mixed with 100 μg/mL Pmb. The mixture was incubated for 30 min at 37°C, followed by ultrafiltration to eliminate unbound Pmb before Zeta potential determination. As controls, the Zeta potential of a solution of 100 μg/mL polymyxin B was determined (Pmb solution). This solution was ultrafiltered to eliminate unbound polymyxin B (Filtered Pmb). **(D)** The same concentrations of OMVs used in **(C)** were analyzed in terms of their Zeta potential. In this case, no polymyxin B was added. For all cases, the experiments were conducted with *n* = 3, and statistical analysis employed a Kruskal-Wallis test with Dunn’s *post hoc* test. Significance levels are denoted as follows: ^*^p < 0.05; ^**^p < 0.01; ^***^p < 0.001. The symbol × in each box represents the media, while dots represent atypical values. Furthermore, 0.25× Amp is a concentration equivalent to one-fourth of the MIC of ampicillin, i.e., 1.56 μg/mL.

The function of OMVs as reservoirs for Pmb, reducing the unbound concentration of the antibiotic, has been previously documented in OMVs from *Escherichia coli* and *Pseudomonas aeruginosa*, as well as in some *S.* Typhi mutants (Kulkarni et al., 2015; Roszkowiak et al., 2019; Marchant et al., 2021). The Zeta potential determination is a valuable method for assessing the interaction between bacterial membranes (or OMVs) and Pmb. As Pmb is a cationic peptide, its interaction with OMVs that have a negative Zeta potential results in depolarization, which can be utilized to evaluate the sequestration of Pmb (Halder et al., 2015; Roszkowiak et al., 2019; Marchant et al., 2021). To assess the sequestering abilities of OMVs generated in the presence of sub-lethal concentrations of β-lactam antibiotics, we measured their Zeta potential and monitored the depolarization caused by the interaction with Pmb. To achieve this, we combined 50 μg/mL of OMVs (calculated based on the protein content) generated by *S.* Typhi cultured in LB (No Amp) or in LB with added ampicillin (0.25× Amp) with various concentrations of Pmb (ranging from 0 to 100 μg/mL). The solution was then kept at 37°C for 30 min, after which it underwent ultrafiltration to remove any unbound polymyxin B before analyzing the Zeta potential of the OMVs. Figure 5A illustrates the response of OMVs obtained in the absence of ampicillin (No Amp), while Figure 5B depicts the outcomes of OMVs obtained with sub-lethal amounts of ampicillin (0.25× Amp). In both scenarios, we noticed a depolarization of OMVs based on the concentration of Pmb. However, there was significant depolarization in OMVs obtained without ampicillin only at higher levels of Pmb (i.e., 50 μg/mL). In contrast, with OMVs from cultures pre-treated with sub-lethal concentrations of ampicillin, the depolarization was noticeable even with lower concentrations of Pmb (i.e., 5 μg/mL).

To independently assess the ability of OMVs to sequester Pmb, we replicated the previous experiment with modifications. This time, we kept the Pmb concentration constant (100 μg/mL) and varied the concentrations of OMVs based on their protein content. For control purposes, we assessed the Zeta potential of a 100 μg/mL Pmb solution, with an expected positive value due to the cationic nature of the Pmb (Pmb solution, Figure 5C). Following this, we performed ultrafiltration on this Pmb solution to eliminate any unbound polymyxin B and then reassessed the Zeta potential of OMVs after resuspending them in ultrapure water. Our results show that this procedure successfully eliminated Pmb from the solution in the absence of OMVs, as evidenced by a nearly complete depolarization of the Zeta potential (Filtered Pmb, Figure 5C). On the other hand, an examination of the Zeta potential of OMVs exposed to Pmb revealed that OMVs derived from a medium containing sub-lethal levels of Amp exhibited a notably more positive Zeta potential (i.e., these OMVs sequester more Pmb) than those obtained from a medium without Amp (Figure 5C). Figure 5D shows the Zeta potential of the same concentrations of OMVs used in Figure 5C, but in the absence of Pmb, no significant differences in the depolarization are shown. All these results indicate that OMVs from a culture supplemented with Amp have a stronger affinity for Pmb. These findings align entirely with the observations in Figures 1, 2. Therefore, OMVs produced by a culture with sub-lethal concentrations of Amp are more effective in sequestering polymyxin B because Amp promotes higher OMV production, with OMVs showing enhanced affinity for Pmb.

**Figure 6:**
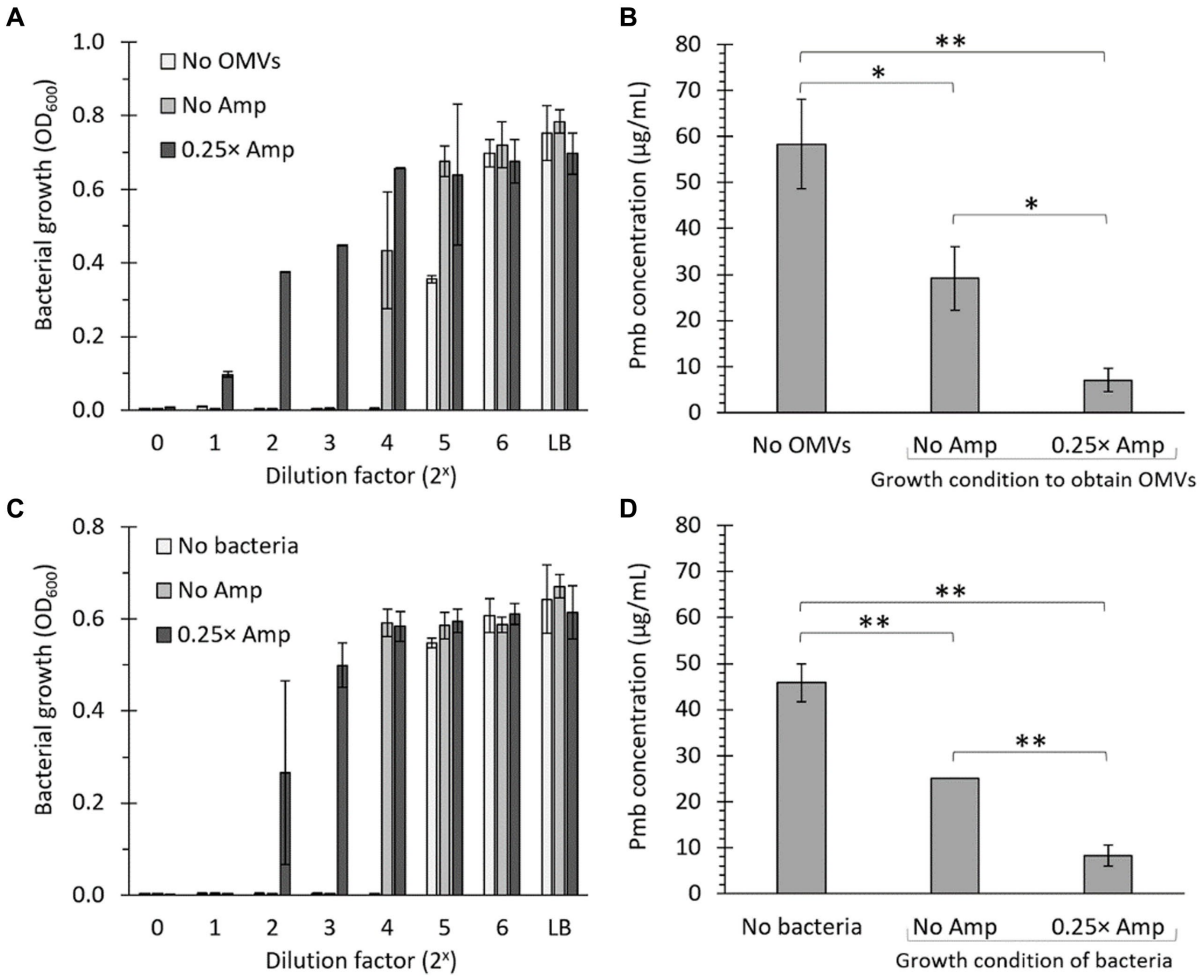
The presence of OMVs decreases the biologically effective concentration of polymyxin B. **(A)** 100 μg/mL Pmb were combined with 50 μg/mL OMVs obtained from *Salmonella* Typhi cultured in LB (No Amp) or LB supplemented with a sub-lethal concentration of ampicillin (0.25× Amp). The mixture underwent a 30-min incubation at 37°C with gentle shaking, followed by ultrafiltration to eliminate OMVs before assessing the growth inhibition of the supernatant. The supernatant was then diluted 10 times in LB (shown as 2^0^ on the x-axis) and subjected to serial dilutions before being seeded with *S.* Typhi as a bioindicator for detecting Pmb (Bacterial growth [OD_600_]). As a control, bacteria were seeded in LB. The experiment was conducted three times. **(B)** The estimation of polymyxin B levels in the ultrafiltrate was established by identifying the final dilution that inhibited the growth of *S.* Typhi, which was considered to have 0.315 μg/mL Pmb. Subsequently, calculations were conducted to ascertain the concentration of Pmb present in the ultrafiltrate. For control purposes, a solution with an identified concentration of 50 μg/mL polymyxin B that had not been mixed with OMVs was utilized (No OMVs). **(C)** As in panel **(A)**, but using 10^7^ CFU/mL of bacteria to retain Pmb instead of OMVs. **(D)** As in panel **(B)**, but using 10^7^ CFU/mL of bacteria to retain Pmb instead of OMVs. 0.25× Amp is a concentration equivalent to one-fourth of the MIC of ampicillin, i.e., 1.56 μg/mL.

To evaluate the capacity of OMVs to reduce the biologically effective concentration (i.e., the antibiotic activity) of Pmb, we combined OMVs (50 μg/mL) with 100 μg/mL Pmb and incubated the mixture for 30 min at 37°C with gentle agitation. Afterward, we removed the OMVs by ultrafiltration before estimating the antibiotic activity of Pmb in the supernatant. The ultrafiltered supernatant was diluted 10-fold in LB, serving as the initial solution (dilution factor 2^0^ in Figures 6A,C). This initial solution underwent serial 2-fold dilutions in LB, and its capacity to hinder the growth of a reporter strain (i.e., *S.* Typhi) was assessed. The higher the dilution required to ascertain the growth inhibition threshold of the reporter strain, the higher the polymyxin B (Pmb) concentration detected in the initial solution. As shown in Figure 6A, when no OMVs were introduced, a 2^4^-fold dilution of the initial solution was necessary to obtain the maximum growth inhibition point of the reporter strain. Conversely, when OMVs extracted from a culture without ampicillin treatment (No Amp) were added, a 2^3^-fold dilution of the initial solution was sufficient to identify the maximum inhibition point of the reporter strain, showing that the presence of OMVs decreased the biologically effective concentration of Pmb. Furthermore, when OMVs derived from a culture treated with sublethal concentrations of ampicillin (0.25× Amp) were introduced, there was no need to dilute the initial solution to ascertain the maximum inhibition point of the reporter strain. This observation shows that the biologically effective concentration of Pmb in the latter solution was the lowest, plausibly because OMVs obtained with ampicillin-sublethal concentrations possess the highest capacity for Pmb sequestration.

To semiquantitatively estimate the biologically effective concentration of Pmb in a solution treated with OMVs, we utilized the data from Figure 6A. The highest dilution that inhibited the growth of the *S.* Typhi reporter strain was deemed the MIC of Pmb, i.e., 0.315 μg/mL. Subsequent calculations were carried out to determine the actual concentration of polymyxin B present in the original solution after the treatment with OMVs. To serve as a control, a solution with a known concentration of 50 μg/mL Pmb that had not been mixed with OMVs was utilized (No OMVs). As shown in Figure 6B, OMVs are able to decrease the biologically effective concentration of Pmb. However, this decrease is much more notorious with OMVs obtained after incubation with sublethal concentrations of ampicillin compared to OMVs obtained with LB alone. The findings align with those depicted in Figure 5, suggesting that the enhanced reduction in the biologically effective concentration of Pmb resulting from OMVs derived from cultures exposed to sub-lethal levels of ampicillin is attributed to a greater capacity for capturing polymyxin B, as compared to OMVs obtained solely from LB.

One of the most challenging parts of working with OMVs is standardizing to compare the biological effects among OMVs extracted from bacteria cultured under different conditions. This issue has been widely acknowledged in the field, although standardization with protein content is generally accepted (McBroom et al., 2006; Deatherage et al., 2009). In this context, we examined the ability of the bacteria to reduce the biologically effective concentration of Pmb. Since bacteria are easily quantifiable even under varying culture conditions, and OMVs typically inherit their envelope properties, it is anticipated that if the presence of β-lactam antibiotics influences the bacterial envelope in a manner conducive to diminishing polymyxin bioactivity, this characteristic will be conveyed to OMVs, as these vesicles originate from evaginations of the bacterial envelope. In this context, we cultured bacteria (*S.* Typhi) in LB without ampicillin or in LB with sub-lethal concentrations of ampicillin, washed them, and standardized their concentration (approximately 10^7^ CFU/mL). Consequently, we repeated the same experiments described in Figures 6A,B but using bacteria instead of OMVs to evaluate the decrease in the biologically effective concentration of Pmb. As expected, similar conclusions were obtained when estimating the sequestration of polymyxin B (Pmb) by determining the reduction in its biologically effective concentration using bacteria instead of OMVs (Figures 6C,D). The results from the experiments using bacteria instead of OMVs further support the findings about the enhanced ability of OMVs obtained from cultures exposed to sub-lethal levels of ampicillin in reducing Pmb concentration through sequestration, compared with OMVs from bacteria not previously exposed to ampicillin.

## Discussion

Our study revealed that sub-lethal doses of β-lactam antibiotics enhance bacterial survival against polymyxin B in *S.* Typhi. This effect arises from β-lactam antibiotics inducing hypervesiculation of OMVs with a higher affinity for polymyxin B, thereby capturing and reducing its biologically effective concentration. As depicted in Figure 1, sub-lethal doses of ampicillin improve bacterial survival against polymyxin B. However, prolonged exposure to sub-lethal doses (i.e., 2 h) further enhances defense against polymyxin B. This phenomenon could be due to an increase in bacterial mass [considering *S.* Typhi STH2370 WT’s generation time of approximately 27 min (Marchant et al., 2021)], as the concentration of ampicillin used (i.e., 0.25× MIC corresponding to 1.56 μg/mL) did not affect significantly bacterial growth. Previous studies have shown that the quantity of bacteria positively correlates with antimicrobial tolerance with different antibiotic families (Kaplan and Koch, 1964; Frenkel et al., 2021), supporting this assertion. Furthermore, enhanced tolerance to polymyxin B is also linked to OMVs, as explained in prior studies where the protective impact was observed after vesicle buildup following incubation for 1 or 2 h within a bacterial community (Marchant et al., 2021). In line with the above claim, Figure 2 showed that washing significantly impacted the MIC of polymyxin B in bacteria, reducing it and supporting the role of OMVs. Furthermore, the enhanced tolerance to polymyxin B from these washed cultures can be regained by including diluted supernatants (5% v/v) and purified OMVs, confirming that vesicles mediate the protection against polymyxin B. To our understanding, the involvement of OMVs in the tolerance to polymyxin B has already been proven for *Escherichia coli*, *Acinetobacter baummanni*, *Haemophilus influenzae*, and *Pseudomonas aeruginosa* (Grenier et al., 1995; Kobayashi et al., 2000; Manning and Kuehn, 2011; Roszkowiak et al., 2019; Park et al., 2021). However, in most cases, the assessment of the influence of these OMVs on polymyxin B tolerance has been examined by introducing purified vesicles to the bacterial culture and then determining the survival level against polymyxin B. Thus, the role of OMVs as defense agents against polymyxin B under more “physiological conditions” (i.e., seeing the effect directly in a culture without the need to add purified OMVs) has been less explored. In this context, it was recently reported that *S.* Typhi hypervesiculatory mutants can protect wild *S.* Typhimurium WT against polymyxin B in a co-culture, demonstrating the role of OMVs directly in the bacterial community without the need to add purified OMVs (Marchant et al., 2021). In this sense, the current research showed that sub-lethal levels of β-lactam antibiotics prompt the generation of OMVs with improved abilities to sequester polymyxin B. This phenomenon can be demonstrated by incorporating purified OMVs from cultures exposed to β-lactam or even by adding diluted supernatant from previously treated cultures with β-lactam antibiotics. Nevertheless, this effect is also noticeable when simply introducing β-lactam antibiotics and polymyxin B to bacterial cultures without directly adding purified vesicles, showing that this phenomenon can plausibly occur in nature, emphasizing the relevance of this study.

The alteration of OMV biogenesis due to the presence of β-lactam antibiotics has previously been documented. In *Klebsiella pneumoniae*, imipenem (a type of β-lactam antibiotic) boosts the release of OMVs. The imipenem-induced OMVs carry distinct contents that increase phagocytosis by macrophages, resulting in pyroptosis and the release of proinflammatory cytokines (Ye et al., 2021). Furthermore, exposure to β-lactam antibiotics results in a higher release of OMVs by *Stenotrophomonas maltophilia* and *Escherichia coli* (Devos et al., 2016; Michel et al., 2020), indicating that the presence of β-lactam antibiotics influences OMV formation not only in *S.* Typhi but across other bacterial species as well. The precise molecular mechanisms through which β-lactam antibiotics stimulate a higher generation of OMVs with different properties than those produced without antibiotic influence are not entirely understood. However, various environmental conditions like temperature, nutrient availability, growth status, quorum sensing, and envelope-targeting antibiotics can modulate levels of vesiculation (Kulp and Kuehn, 2010; Roier et al., 2016). Additionally, alterations in the peptidoglycan composition and structure, along with the accumulation of peptidoglycan fragments, have been linked to increased OMV production (Kulp and Kuehn, 2010; Schwechheimer et al., 2013, 2014; Pasqua et al., 2021). In this context, it has been documented that deleting genes directly or indirectly related to cell wall integrity affects the formation of OMVs. This includes changes in the quantity and quality of the produced vesicles (Nevermann et al., 2019; Li et al., 2022). For instance, PBP-1b (penicillin-binding protein encoded by *mrcB*) is a dual-function enzyme (with transglycosidase and transpeptidase activity) involved in peptidoglycan synthesis (Sauvage et al., 2008; Kumar et al., 2012). In this sense, *S.* Typhi Δ*mrcB* displays increased vesicle production with altered protein content compared to the parental strain, highlighting the importance of the bacterial cell wall in vesiculation (Nevermann et al., 2019). Nevertheless, although it is well known that β-lactam antibiotics block the activity of penicillin-binding proteins (PBPs), inhibiting cell wall synthesis, it has been reported that these antibiotics also produced other alterations in bacterial cells. For instance, sub-lethal concentrations of β-lactam antibiotics induce the RpoS (sigma S) regulon, which is involved in the general stress response in bacteria. This induction is observed in *Escherichia coli*, *Vibrio cholerae*, and *Pseudomonas aeruginosa* (Gutierrez et al., 2013). Since the RpoS regulon is extensive and encompasses a wide range of genes involved in various physiological functions (Lago et al., 2017), it is plausible that conditions affecting this regulon could also be contributing to the altered OMV phenotype observed in *S.* Typhi upon treatment with sub-lethal concentrations of β-lactam antibiotics. In addition, β-lactams, even at concentrations below the MIC, have pronounced effects on the quantitative composition of the outer membrane of *Escherichia coli*, including changes in the phospholipid/amino acid ratio and the lipopolysaccharide/amino acid ratio, also increasing the amount of peptidoglycan fragments bound to the outer membrane; albeit the precise link between the primary mechanisms of action of the antibiotics and these secondary effects is unknown (Suerbaum et al., 1987). According to this evidence, β-lactam antibiotics exert multiple effects on bacterial cells, strongly suggesting that the mechanism by which they affect vesiculation is multifactorial. This would explain the alterations we observed for OMV biogenesis after treatment with sub-lethal concentrations of beta-lactams, including changes in the OMV size. The distinct protein profile of the OMVs collected in ampicillin’s presence compared to those obtained without antibiotics underscores its influence on OMV biogenesis.

The Zeta potentials of OMVs from cultures with and without sublethal levels of β-lactam antibiotics shed light on the intriguing variations in their affinity for polymyxin B. This highlights the potential of ampicillin to influence the physical properties and behavior of OMVs, particularly regarding their interactions with polymyxin B. Moreover, the results obtained from the assessment of polymyxin B concentration in the supernatant after incubation with OMVs further emphasize the enhanced capacity of OMVs from cultures exposed to sublethal ampicillin concentrations to reduce the bioactivity of polymyxin B. This same strategy was previously reported to assess the sequestering ability of OMVs derived from *S.* Typhi Δ*tolR* mutants (Marchant et al., 2021). The capability of OMVs to sequester polymyxin B and mitigate its antibacterial effects stands as a pivotal discovery in comprehending the adaptive responses of bacteria to antibiotic exposure.

Polymyxin functions by interacting with the bacterial outer membrane through electrostatic and hydrophobic interactions (Velkov et al., 2010; Trimble et al., 2016). Nevertheless, evidence also suggests that polymyxin interacts with outer membrane proteins (van der Meijden and Robinson, 2015). In this study, we found that OMVs obtained after treatment with a sub-lethal dose of β-lactam antibiotics exhibit a higher sequestering ability of polymyxin B compared to those obtained without antibiotics despite having similar Zeta potential. A similar result was observed in OMVs from the *S.* Typhi Δ*tolR* mutant, which showed increased sequestering activities than the OMVs from *S.* Typhi WT, although their Zeta potential was similar (Marchant et al., 2021). Our findings indicate that the increased protective capacity of OMVs may be mainly due to hydrophobic interactions, as previously suggested (Marchant et al., 2021). The implications of these findings offer a deeper understanding of the adaptive responses of bacteria to antibiotics and the potential role of OMVs in modulating antibiotic effectiveness.

The inappropriate use of antibiotics can yield various consequences. Misuse and overuse of antimicrobials contribute to alterations in the microbiota, and the emergence and dissemination of multidrug-resistant organisms, among other consequences, resulting in heightened morbidity, mortality, and the utilization of finite patient-care resources (Becattini et al., 2016; Dyar et al., 2017; Piltcher et al., 2018). While these effects are widely acknowledged, the correlation between antibiotic misuse and its repercussions dependent on OMVs has recently garnered attention. For instance, exposure to β-lactam antibiotics induces an increased release of OMVs by *Stenotrophomonas maltophilia*, which are laden with β-lactamases (Devos et al., 2016). These β-lactamase-packed OMVs exhibit the capability for extracellular β-lactam degradation. Derived from *Stenotrophomonas maltophilia*, these OMVs significantly elevate the apparent MICs of imipenem and ticarcillin for cohabitating species such as *Pseudomonas aeruginosa* and *Burkholderia cenocepacia* (Devos et al., 2016). Notably, the OMVs confer protection to the β-lactamases against proteases, ensuring their stability and sustained activity over prolonged periods (Devos et al., 2016). On the other hand, the release of Pal (peptidoglycan-associated lipoprotein) from *Escherichia coli* in response to various antibiotics, namely ampicillin, gentamicin, and levofloxacin, has been reported (Michel et al., 2020). The findings indicate that ampicillin significantly increases the release of Pal compared to the other antibiotics under examination (Michel et al., 2020). The authors postulate that most Pal is discharged within OMVs, which also encapsulates lipopolysaccharides (LPS) and various outer membrane and periplasmic proteins (Michel et al., 2020). The presence of Pal and other *Escherichia coli* proteins in the OMVs was validated through Western blot analysis (Michel et al., 2020). That study posits that releasing Pal and LPS in OMVs induced by specific β-lactam antibiotics, such as ampicillin, might contribute to the excessively robust inflammatory response observed in bacterial sepsis (Michel et al., 2020). Furthermore, in *Klebsiella pneumoniae*, imipenem amplifies the secretion of OMVs characterized by an elevated presentation of GroEL, a protein that enhances the phagocytosis of OMVs by macrophages through interaction with the receptor LOX-1 (Ye et al., 2021). These OMVs induce pyroptosis of macrophages and the subsequent release of proinflammatory cytokines, culminating in exacerbated inflammatory responses and increased mortality in infected mice (Ye et al., 2021).

In this study, we showed the enhanced protective capabilities of OMVs against Pmb, driven by sub-lethal doses of β-lactams, underscoring the potential role of OMVs in mediating antibiotic tolerance, independent of genetic determinants. Nevertheless, it is plausible to speculate that β-lactam antibiotics could also contribute to developing resistance to polymyxin B via OMVs in a bacterial community via genetic determinants (e.g., mutations). From an evolutionary perspective, antibiotics can be considered as a stressor. As the stress intensifies (i.e., with higher antimicrobial concentrations), mutations conferring a sufficiently strong resistance phenotype become less frequent (Orencia et al., 2001; Quinones-Soto and Roth, 2011). Conversely, as the stress diminishes (i.e., with lower antimicrobial concentrations), selection pressures relax, and mutations causing subtle phenotypic changes that can enable bacterial replication are observed more frequently (Orencia et al., 2001; Quinones-Soto and Roth, 2011). These “subtle” mutations allow bacteria to replicate in a stressful environment with a relaxed selection, representing new opportunities for accumulating more mutations that could contribute, in turn, to increase the resistance/tolerance levels (Orencia et al., 2001; Roth, 2010; Quinones-Soto and Roth, 2011). Therefore, the “strength of the stressors” could modulate bacterial population dynamics by favoring the fixation of new traits (Orencia et al., 2001; Quinones-Soto and Roth, 2011). In other words, elements that reduce antibiotic concentrations could play a crucial role in developing genetically determined resistance mechanisms. Given that sublethal concentrations of β-lactam antibiotics lead to heightened production of OMVs, thereby augmenting their ability to sequester Pmb and reduce its effective biological concentration, it is conceivable to propose that the presence of these OMVs might serve as a mechanism for relaxing selective pressure (i.e., decreasing antibiotic concentration), potentially fostering an environment conducive to the accumulation of mutations associated with resistance or tolerance phenotypes. Nevertheless, experimental validation of this hypothesis is required to assess the potential role of OMVs in bacterial resistance dissemination based on genetic determinants, emerging as a novel field to be explored.

Our results emphasize the complex interactions among different types of antibiotics, underlining the need to assess not only the individual effectiveness of antimicrobials but also their potential secondary impacts on tolerance to other antimicrobial agents. This finding underlines the importance of conducting a more comprehensive evaluation of the clinical and epidemiological implications linked to exposure to β-lactam antibiotics and the role of OMVs in this phenomenon.

In conclusion, our study sheds light on the multifaceted impact of sublethal concentrations of β-lactam antibiotics on OMV production, cargo composition, and their capacity to sequester antimicrobial compounds. These findings offer insights into the potential role of β-lactam antibiotics in modulating the biogenesis and properties of OMVs in *Enterobacteriaceae*. The subsequent phase of this research could entail further exploration into the specific mechanisms through which β-lactam antibiotics induce these changes in OMVs, as well as an examination of the functional implications arising from the altered properties of OMVs. Moreover, assessing the impact of β-lactam antibiotics on the interaction between OMVs and other antimicrobial agents is a crucial target for future inquiry.

## Data availability statement

The raw data supporting the conclusions of this article will be made available by the authors, without undue reservation.

## Author contributions

PM: Writing – review & editing, Methodology, Investigation, Formal analysis, Conceptualization. EV: Writing – review & editing, Methodology, Investigation, Conceptualization. AS: Writing – review & editing, Project administration, Methodology, Investigation. JN: Writing – review & editing, Methodology, Investigation. IF: Writing – review & editing, Methodology, Investigation. BB: Writing – review & editing. CO: Writing – review & editing, Resources, Funding acquisition. IC: Writing – review & editing, Resources, Formal analysis. FG: Writing – review & editing, Resources, Formal analysis. JF: Writing – review & editing, Writing – original draft, Visualization, Validation, Supervision, Resources, Project administration, Methodology, Investigation, Funding acquisition, Formal analysis, Data curation, Conceptualization.
